# TransNeural: An Enhanced-Transformer-Based Performance Pre-Validation Model for Split Learning Tasks

**DOI:** 10.3390/s24165148

**Published:** 2024-08-09

**Authors:** Guangyi Liu, Mancong Kang, Yanhong Zhu, Qingbi Zheng, Maosheng Zhu, Na Li

**Affiliations:** 1 China Mobile Research Institute, Beijing 100053, China; liuguangyi@chinamobile.com (G.L.); zhengqingbi@chinamobile.com (Q.Z.); linawx@chinamobile.com (N.L.); 2 School of Communications and Information Engineering, Beijing University of Posts and Telecommunications, Beijing 100876, China; 3 School of Electronics and Information Engineering, Beijing Jiaotong University, Beijing 100091, China; 4 ZGC Institute of Ubiquitous-X Innovation and Application, Beijing 100191, China; 5 China Mobile (Suzhou) Software Technology Co., Ltd., Suzhou 215163, China; zhumaosheng@cmss.chinamobile.com

**Keywords:** 6G, digital twin network, transformer, split learning, pre-validation environment

## Abstract

While digital twin networks (DTNs) can potentially estimate network strategy performance in pre-validation environments, they are still in their infancy for split learning (SL) tasks, facing challenges like unknown non-i.i.d. data distributions, inaccurate channel states, and misreported resource availability across devices. To address these challenges, this paper proposes a TransNeural algorithm for DTN pre-validation environment to estimate SL latency and convergence. First, the TransNeural algorithm integrates transformers to efficiently model data similarities between different devices, considering different data distributions and device participate sequence greatly influence SL training convergence. Second, it leverages neural network to automatically establish the complex relationships between SL latency and convergence with data distributions, wireless and computing resources, dataset sizes, and training iterations. Deviations in user reports are also accounted for in the estimation process. Simulations show that the TransNeural algorithm improves latency estimation accuracy by 9.3% and convergence estimation accuracy by 22.4% compared to traditional equation-based methods.

## 1. Introduction

In the era of the sixth generation mobile network (6G) [1,2], an increasing number of artificial intelligence (AI) applications will rely on edge network to train their continually expanding models based on massive amounts of user data [3]. While users may be willing to provide their data with payment, they are concerned about data privacy issues. Meanwhile, fully training AI models on user devices to upload only model parameters can address privacy issues, but is often impractical due to constraint user resources [4]. Additionally, companies are reluctant to fully disclose their model parameters, since these are valuable assets [5]. In this context, split learning (SL) has emerged as a promising solution [6]. In “SL for model training process”, an edge server (ES) connected with an access point (AP) offloads only a few lower layers of AI models to a user device for local training, where the device interacts intermediate results in forward and backward propagations with ES to update the upper layers. Once a user device finishes updating using its own data, it sends the updated lower layers to the next user device via AP, continuing this process until all participants have contributed their data. This model training method protects user privacy and conserves device resources, and we abbreviate it as “SL tasks”. The applications include illness model training in healthcare [7] and user behavior analysis in mobile networks [8], etc.

With the adoption of SL technology, a crucial concern for AI application companies is to pre-estimate SL latency and convergence performance under given resource allocation strategy before paying users and operators for data, wireless, and computing resources [6]. However, under unknown non-i.i.d. data distribution [9], inaccurate wireless channel state information (CSI), and misreported available computing resources on different user devices, it becomes extremely difficult to accurately estimate the SL performance before real physical executions. The reason is as follows. Suppose there are three participate user devices: A, B, and C. If the data distribution on A is similar to that on C, but significantly different from B, then the training sequences A-C-B and A-B-C will result in considerable differences in SL training convergence, despite utilizing the same amount of resources. The situation becomes even more problematic when there exists reporting deviations in CSI, amount of data and computing resources on devices. Consequently, AI application companies face high risks when investing heavily in network operators and user devices for SL training on edge, without confidence in achieving their training latency and convergence goals, which significantly dampens their enthusiasm.

The Digital Twin Network (DTN), emerging as a critical 6G technology [10], is an intelligent digital replica of a physical network that synchronizes physical states in real-time and uses AI models to analyze the characteristics and relationships of network components for accurate inferences in 6G network [11]. In the context of SL, DTN is expected to automatically model data similarities between devices, account for user misreporting and correct inaccurate CSI based on the unique profiles of different devices. Moreover, it can learn the complex relationships between service performance and network parameters. These functions can be realized based on AI models and form a pre-validation environment in DTN for testing SL strategy performance. Based on the pre-validation results, DTN can repeatedly optimize the SL strategy until it meets latency and convergence requirements before deployment in the physical world.

Nevertheless, several challenges exist in establishing DTN pre-validation environment for SL training tasks. First, learning data similarities between different physical devices is difficult due to data privacy issues, which prevent the acquisition and analysis of user data. Second, accurately estimating the misreported behaviors of each physical device is challenging. These deviations depend on multiple factors such as device version, user behavior, movement patterns, etc. However, the actual physical information is unattainable due to user privacy, data collection errors, and other factors, making it impossible to learn errors from historical data. Third, it is difficult to model the complex relationships between various SL training performance metrics and network factors like data similarities, dataset size, training iterations, device resources, wireless channel quality, and edge server resources, particularly when unknown parameters exist. Fourth, the model needs to be broadly applicable to various SL requirements (such as latency, convergence, and energy efficiency) and different tasks. This would enable an autonomous modeling process for pre-validation environments, advancing toward the ultimate goal of realizing fully automatic network management in DTNs [12].

Existing AI models for the DTN pre-validation environment can be divided into three categories. First, long short-term memory (LSTM) networks are used to establish DTN models for predicting future states. For example, the authors of [13] used LSTM to build a network traffic prediction model for DTN based on historical traffic data from a real physical network, which can be utilized for network optimization. Similarly, the authors of [14] employed LSTM to model and predict changes in reference signal receiving power, aiming to reduce handover failures in DTN. Second, graph neural network (GNNs) are used to model network topology for strategy pre-validation. For instance, the authors of [15] designed a GNN-based pre-validation environment for DTN to estimate delay and jitter under given queuing and routing strategies. Likewise, the authors of [16] proposed a GNN-based pre-validation model to estimate end-to-end latencies for different network slicing schemes. Third, a combination of GNN and transformer models is used to capture both temporal and spatial relationships among network components. For example, the authors of [17] developed a DTN pre-validation environment that predicts end-to-end quality of service for resource allocation strategies in cloud-native micro-service architectures.

Although existing methods are important explorations for building an intelligent DTN pre-validation environment, several problems persist in the context of SL training tasks. First, LSTM and GNN-based models are only adept at learning the relationships between adjacent nodes, where the learning performance drops quickly as the device separation grows. This property makes them suitable for scenarios where only the relationships between adjacent nodes are critical, such as time-series predictions [18]. However, quickly learning the data relationships between all devices is crucial for accurate SL performance estimation, since every device has the potential to participate with a random sequence. Second, while transformer-based models excel in learning inter-node relationships, they may struggle with understanding the relationships within the feature vector of a single node. This capability is vital for accurately modeling and predicting the performance of SL training tasks in wireless network.

Based on these considerations, this paper presents a TransNeural algorithm for DTN pre-validating environment towards SL training tasks. This algorithm integrates transformers with neural network, leveraging the transformer’s strength in inter-node learning [19] to capture data similarities among different user devices and the neural network’s strength in inter-feature learning [20] to understand the complex relationships between various system variables, SL training performances, and deviation characteristics between different nodes. The contributions of this work can be summarized as follows:

- A mathematical model for SL training latency and convergence is established, which jointly considers unknown non-i.i.d. data distributions, device participate sequence, inaccurate CSI, and deviations in occupied computing resources. These are crucial factors for SL training performance, but are often overlooked in existing SL studies.
- To close the gap in SL-target DTN pre-validation environment, we propose a TransNeural algorithm to estimate SL training latency and convergence under given resource allocation strategies. This algorithm combines the transformer and neural network to model data similarities between devices, establish complex relationships between SL performance and network factors such as data distributions, wireless and computing resources, dataset sizes, and training iterations, and learn the reporting deviation characteristics of different devices.
- Simulations show that the proposed TransNeural algorithm improves latency estimation accuracy by 13.44% compared to traditional equation-based algorithms and enhances convergence estimation accuracy by 116.74%.

The remainder of our work is organized as follows. Section 2 gives the system model. Section 3 designs the TransNeural algorithm in DTN for estimating the latency and convergence of SL training tasks. Simulation results and discussions are given in Section 4. Finally, we conclude this paper in Section 5.

## 2. System Description

In this section, we sequentially present the system model, communication model, SL process and DTN.

### 2.1. System Model

**Figure 1:**
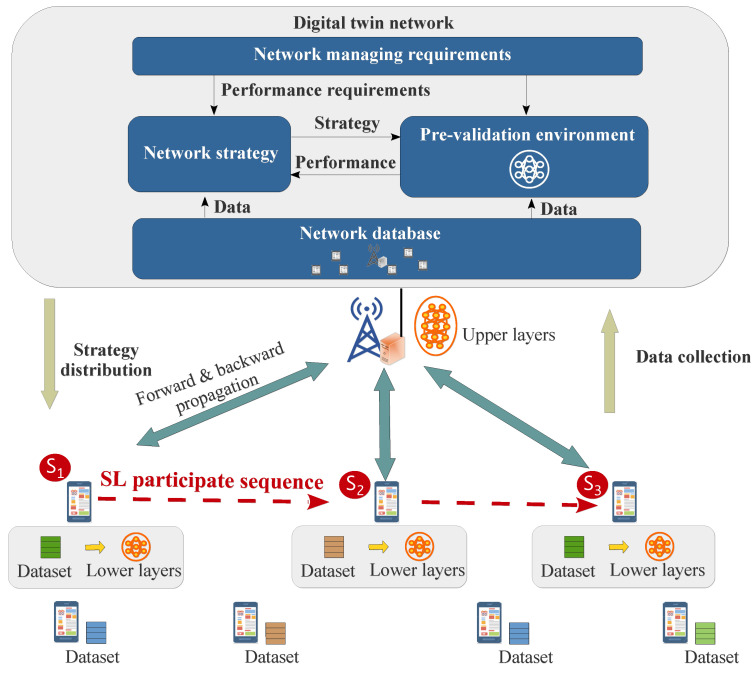
DTN-integrated network for SL training tasks.

Figure 1 gives the whole system of DTN-integrated network for SL training tasks. In our system, there are *N* user devices access to an AP equipped with an edge server (ES). Each device is characterized by some unique features, including data distributions, the amount of available computing resources, misreporting behaviors, etc. On the *n*-th device, the dataset is denoted as $Q_{n}$ with total size $Q_{n}^{\mathrm{sum}}=\left|\right|Q_{n}\left|\right|$; the amount of available computing resource is denoted as $f_{n}$. The data similarities between different devices depend on their users habits (such as shopping habits) and behaviors. The distance between the *n*-th device with AP is denoted as *d*. The index of key notations are listed in Table 1.

There is a DTN deployed on the ES, denoted as D. It synchronizes network states via AP, and generates network strategies (such as resource allocation strategies) according to requirements of oncoming tasks (such as SL training tasks). Specifically, it has a pre-validation environment for testing and optimizing strategies before they are executed in the real physical network, forming a closed-loop strategy optimization in DTN. The contribution of our work is concentrated on the establishment of the pre-validation environment for SL training tasks.

In SL training process, a target AI model is partitioned into “upper layers” and “lower layers”. Under a given resource allocation strategy, the AP would sequentially offload the lower layers to the participate devices, which are responsible for computing forward and backward propagation for lower layers. The updated lower layers would be transmitted to the next participate device via AP until all participants have contributed and the final updated lower layers will be sent back to ES to merge with upper layer. This process can train the target AI model while protecting user data privacy and conserving device resources.

### 2.2. Communication Model

The AP is equipped with *M* antenna, and each user device is equipped with one antenna. The transmission rate of each user device is

(1) $$\begin{array}{c} R_{n,i}=B_{n}\cdot \log_{2}\left(1+\frac{P_{n,i,t}\cdot H_{n,t}}{N_{0}B_{n}}\right), \end{array}$$

where $B_{n}$ is the allocated bandwidth for the *n*-th device, i={up,dn} represent uplink and downlink, $P_{n,i,t}$ is the instant uplink or downlink transmitting powers between the *n*-th device and AP in the *t*-th time slot, $N_{0}$ is the power spectral density of noise, $H_{n,t}=L_{n}\cdot h_{n,t}$ is the instant channel gain, where $L_{n}$ is large-scale fading and $h_{n,t}$ is small-scale fading. The channel gain $H_{n,t}$ is a circularly symmetric complex Gaussian with zero mean and variance $\sigma_{n}^{2}$ [21], which randomly changes across different time slots. The probability density functions (PDF) of $H_{n,t}$ in uplink and downlink are given in Theorem 1.

**Theorem** **1** (PDF for SIMO and MISO channels). *The PDF of channel gain for SIMO and MISO can be derived as $u\left(x\right)=\frac{1}{\sigma_{n}^{2}\Gamma \left(M\right)}{\left(x/\sigma_{n}^{2}\right)}^{M-1}e^{-x/\sigma_{n}^{2}}$, according to [22,23].*

To maximize transmission rate and cancel the influence from small-scale channel gain changing, we adopt a small-scale power adaptation scheme from [24] on each time slot. That is, the uplink allocated transmitting power $P_{n}$ of device *n* and the downlink transmitting power $P_{\mathrm{AP}}$ of AP are taken as the uplink and downlink average power between different time slots. The scheme adapts the instantaneous power according to the channel gain in the *t*-th time slot by

(2) $$P_{n,i,t}=\left\{\begin{array}{cc} \frac{P_{n,i,\mathrm{tar}}}{H_{n,t}}, & \mathrm{if}\;H_{n,t}\geq H_{L}\\ 0, & \mathrm{else}, \end{array}\right.$$

where $H_{L}$ is the lower bound of channel gain, $P_{n,i,\mathrm{tar}}$ is target receiving power. Take the uplink transmission process of the *n*-th device as an example, the relationship between the target receiving power with the average transmitting power $P_{n}$ is

(3) $$\begin{array}{cc} P_{n} & =E\left[P_{n,\mathrm{up},t}\right]=P_{n,\mathrm{up},\mathrm{tar}}\cdot \int_{H_{L}}^{\infty }\frac{{\left(x/\sigma_{n}^{2}\right)}^{M-1}e^{-x/\sigma_{n}^{2}}}{\sigma_{n}^{2}\Gamma \left(M\right)\cdot x}dx\\ & =\frac{P_{n,\mathrm{up},\mathrm{tar}}}{\sigma_{n}^{2}}\cdot \frac{\Gamma^{u}\left(M-1,H_{L}/\sigma_{n}^{2}\right)}{\Gamma (M)}, \end{array}$$

where $\Gamma^{u}\left(\cdot \right)$ and $\Gamma^{D}\left(\cdot \right)$ are the upper and lower incomplete Gamma functions, respectively. Therefore, the target receiving power at AP and the *n*-th device can be derived as

(4) $$\begin{array}{c} P_{n,\mathrm{up},\mathrm{tar}}=P_{n}\cdot \frac{\Gamma^{u}\left(M-1,H_{L}/\sigma_{n}^{2}\right)}{\sigma_{n}^{2}\cdot \Gamma \left(M\right)},\;P_{n,\mathrm{dn},\mathrm{tar}}=P_{\mathrm{AP}}\cdot \frac{\Gamma^{u}\left(M-1,H_{L}/\sigma_{n}^{2}\right)}{\sigma_{n}^{2}\cdot \Gamma \left(M\right)}. \end{array}$$

By introducing the small-scale power adaptation scheme, the maximized transmission rate is converted from Equation (1) to

(5) $$R_{n,i}=B_{n}\log_{2}\left(1+\frac{P_{n,i,\mathrm{tar}}}{N_{0}B_{n}}\right).$$

### 2.3. Split Learning Process

The SL process trains its target AI model based on a given resource allocation scheme, which chooses a set of user devices to participate the SL training process and decide their working sequence. We use a variable $s_{k}\;\left(k=1,2,\cdots ,K\right)$ to contain the device number, which is assigned as the *k*-th working device participating in the SL training process. Then, the target AI model is split into lower layers $M_{\mathrm{lw}}^{\mathrm{APP}}$ and upper layers $M_{i}^{\mathrm{APP}}$. The bit-size of $M_{\mathrm{lw}}^{\mathrm{APP}}$ is $b_{\mathrm{lw}}$. Then, the lower layer $M_{\mathrm{lw}}^{\mathrm{APP}}$ is offloaded to the device whose number is $s_{1}$. Then, the $s_{1}$-th device extract a mini-batch from its local dataset to perform forward propagation with computing workload $\left(Q_{s_{k}}\cdot c_{\mathrm{lw}}^{\mathrm{forward}}\right)$, where $Q_{s_{k}}$ is the size of a mini-batch, which need to be smaller than the total amount of data $\left(Q_{s_{k}}<Q_{s_{k}}^{\mathrm{sum}}\right)$ on the $s_{k}$-th device. Then, it passes the middle parameters of forward propagation to AP with bit-size $(Q_{s_{k}}\cdot b_{\mathrm{mid},\mathrm{fw}}$), where $b_{\mathrm{mid},\mathrm{fw}}$ is the bit-size of the middle results of one training sample. Based on the received middle parameters, the ES continues to perform forward propagation for upper layers with computing workload $\left(Q_{s_{k}}\cdot c_{\mathrm{up},\mathrm{fw}}\right)$. After that, it computes backward propagation for upper layers with computing workload $\left(Q_{s_{k}}\cdot c_{\mathrm{up},\mathrm{bk}}\right)$. Then, it sends the middle parameter of backward propagation back to user device via AP, with bit-size $\left(Q_{s_{k}}\cdot b_{\mathrm{mid},\mathrm{bk}}\right)$. Next, user device computes backward propagation with computing workload $\left(Q_{s_{k}}\cdot c_{\mathrm{lw},\mathrm{bk}}\right)$. This process iterates $I_{k}$ times. After that, the $s_{k}$ user device would send the updated lower layers to AP, which sends them to the next participate user $s_{k+1}$ to continue SL training process, until all participants has trained the target AI model.

### 2.4. Digital Twin Network

The DTN can be model as

(6) $$\begin{array}{c} D=\left\{O,G,E,Z\right\}, \end{array}$$

where O,G,E,Z are network management module, network strategy module, pre-validation environment and network database, respectively. Among them, the network management module O is responsible for analyzing the requirements of different tasks (such as SL tasks), and orchestrating different modules to reach the requirements. Network strategy module G can generate resource allocation solutions based on intelligent algorithms (such as deep reinforcement learning algorithms, generative AI algorithms, etc.), whose performance can be tested in the pre-validation environment E, and based on the testing results, the module G can improve its strategy. The design of network strategy module is beyond the scope of this work. Specifically, this paper focus on the establishment of pre-validation environment E, which needs to estimate the performance of a resource allocation solution for SL training tasks, which is essential to guarantee the SL performance. Its input includes current network states and resource allocation solution; its output is the SL performance metrics such as latency and convergence. Notice that the estimating process is challenge because of unknown data distributions and misreported wireless channel qualities and available computing resources. Finally, network database Z can be expressed as

(7) $$\begin{array}{c} Z=\left\{f_{\mathrm{av},n}^{*},L_{n}^{*},||Q_{n}\left|\right|,f_{\mathrm{AP}}\right\}, \end{array}$$

where the superscript “*” represents that the recorded data in the DTN has some deviation from the actual data. $L_{n}^{*}$ is the recorded large-scale fading of the *n*-th device in the DTN, corresponding to a deviated root square variant $\sigma_{n}^{*}$ of channel gain. Notice that the DTN does not need to collect the small-scale fading, since we deploy a small-scale power adaptation scheme on user devices and AP to deal with it in Section 2.2. $f_{\mathrm{av},n}^{*}$ is the recorded available amount of computing resources of the *n*-th device in the DTN, which may have error from the real amount of computing resources $f_{\mathrm{av},n}$. The error becomes from user misreported behavior, data collecting error, and sudden appeared emergency tasks. Under a computing resource allocation solution $f_{n}$ generated by network strategy module G, the actual occupied computing resource on the *n*-th device is inaccurate compared with the allocated computing resource $f_{n}$, which can be expressed as

(8) $$\begin{array}{c} f_{n}^{*}=\min\left\{f_{\mathrm{av},n}^{*},f_{n}\right\}. \end{array}$$

## 3. Transformer-Based Pre-Validation Model for DTN

The aim of this paper is to establish a pre-validation environment for estimating latency and convergence of SL training tasks under given network states and strategies. To do this, this section first analyzes the relationships between the estimation objects, i.e., SL training latency and convergence, with network states and strategies from mathematical view, where several unknown parameters exist and makes the estimation process difficult. To cope with the difficulties, we propose a TransNeural algorithm which combines transformer and neural network to learn inter-nodes and inter-feature relationships with unknown parameters.

### 3.1. Problem Analysis for SL Convergence Estimation

The SL training process comprises iterative wireless transmission process and computing process on AP and different devices. Without causing ambiguity, we use divergence ε to replace convergence (1−ε). First, the wireless outage can cause transmission failure in middle parameter forward and backward propagations. Based on the small-scale power adaptation scheme in Section 2.2 and PDF of channel gain in Theorem 1, the outage probability in different wireless transmission process can be expressed as

(9) $$\begin{array}{c} \varepsilon_{s_{k},\mathrm{tr}}^{*}=\int_{0}^{H_{L}}\frac{{\left(x/{\left(\sigma_{s_{k}}^{*}\right)}^{2}\right)}^{M-1}}{{\left(\sigma_{s_{k}}^{*}\right)}^{2}\cdot \Gamma \left(M\right)}e^{-x/{\left(\sigma_{s_{k}}^{*}\right)}^{2}}dx=\frac{\Gamma^{D}\left(M,H_{L}/{\left(\sigma_{s_{k}}^{*}\right)}^{2}\right)}{\Gamma (M)}. \end{array}$$

The outage probability is inaccurate because the root square variant $\sigma_{n}^{*}$ of channel gain is inaccurate. Because of outage probability in uplink and downlink, the usable amount of data in a mini-batch in one iteration changes from $Q_{s_{k}}$ to $\left({\left(1-\varepsilon_{s_{k},\mathrm{tr}}^{*}\right)}^{2}\cdot Q_{s_{k}}\right)$.

In addition, the original divergency rate in computing process can be expressed as

(10) $$\begin{array}{c} \varepsilon_{\mathrm{cp}}^{⋎}=\exp\left(-\mu^{⋎}QI\right), \end{array}$$

where $\mu^{⋎}$ is an unknown divergence parameter depending on the data distribution on different devices. The superscript “⋎” denotes that the value of a variable is unknown. Then, considering outage probability in wireless transmission process, the finally divergence on $s_{k}$-th device is

(11) $$\begin{array}{c} \varepsilon_{s_{k}}^{⋎}=\exp\left(-\mu_{s_{k}}^{⋎}\cdot {\left(1-\varepsilon_{s_{k},\mathrm{tr}}^{*}\right)}^{2}\cdot Q_{s_{k}}\cdot I_{s_{k}}\right). \end{array}$$

The overall training divergence throughout different devices needs to consider data similarity among different devices, which can be expressed with an unknown data correlation matrix

(12) $$\begin{array}{c} C^{⋎}=\left[\begin{array}{cccc} C_{1,1}^{⋎} & \cdots & C_{1,N-1}^{⋎} & C_{1,N}^{⋎}\\ C_{2,1}^{⋎} & \cdots & C_{2,N-1}^{⋎} & C_{2,N}^{⋎}\\ \vdots & \begin{array}{c} ⋰ \end{array} & \vdots & \vdots \\ C_{N,1}^{⋎} & \cdots & C_{N,N-1}^{⋎} & C_{N,N}^{⋎} \end{array}\right], \end{array}$$

where $C_{k}^{⋎}=\left[C_{k,1}^{⋎},C_{k,2}^{⋎},\cdots ,C_{k,N}^{⋎}\right]$ is the data similarity vector of device *k*. Then, the overall divergence can be expressed as

(13) $$\begin{array}{c} \varepsilon_{\mathrm{total}}^{⋎}=\exp\left(-\left(\sum_{k=1}^{K}{\left(1-\varepsilon_{s_{k},\mathrm{tr}}^{*}\right)}^{2}\mu_{s_{k}}^{⋎}Q_{s_{k}}I_{s_{k}}-\sum_{k=1}^{K-1}{\left(1-\varepsilon_{s_{k},\mathrm{tr}}^{*}\right)}^{2}C_{s_{k},s_{k+1}}^{⋎}Q_{s_{k}}I_{s_{k}}\right)\right). \end{array}$$

### 3.2. Problem Analysis for SL Latency Estimation

The latency of SL training process is the sum of transmitting and computing latencies on AP and different participate devices. In detail, first, the lower layers $M_{\mathrm{lw}}^{\mathrm{APP}}$ with bit-size $b_{\mathrm{lw}}$ is wirelessly transmitted to $s_{k}$-th device, where the downlink transmission latency is

(14) $$\begin{array}{c} D_{s_{k},\mathrm{tr},\mathrm{ofld}}^{*}=b_{\mathrm{lw}}/R_{n,\mathrm{dn}}^{*}, \end{array}$$

where the transmission rate is inaccurate. The reason is that the root square variant $\sigma_{n}^{*}$ of channel gain is inaccurate, which leads the calculated target power $P_{n,\mathrm{dn},\mathrm{tar}}$ to be inaccurate according to Equation (4); thus, the transmission rate $R_{n,\mathrm{dn}}^{*}$ is inaccurate according to Equation (5). Then, the $s_{k}$-th user device computes forward propagation with computing latency

(15) $$\begin{array}{c} D_{s_{k},\mathrm{lc},\mathrm{fw}}^{*}=\frac{Q_{s_{k}}\cdot c_{\mathrm{lw},\mathrm{fw}}}{f_{s_{k}}^{*}}, \end{array}$$

where $f_{s_{k}}^{*}$ is actual allocated computing resources on the $s_{k}$-th device, which is inaccurate according to Equation (8). Then, the middle results of forward propagation is transmitted to AP with uplink transmission latency

(16) $$\begin{array}{c} D_{s_{k},\mathrm{tr},\mathrm{fw}}^{*}=\frac{Q_{s_{k}}\cdot b_{\mathrm{mid},\mathrm{fw}}}{R_{n,\mathrm{up}}^{*}}. \end{array}$$

The AP performs forward propagation for the upper layers using the accepted middle data. Then, it computes the loss function and performs backward propagation for the upper layers. The total computing latency of this forward and backward propagation process is

(17) $$\begin{array}{c} D_{s_{k},\mathrm{AP}}^{*}=\frac{\left(1-\varepsilon_{s_{k},\mathrm{tr}}^{*}\right)\cdot Q_{s_{k}}\cdot \left(c_{\mathrm{up},\mathrm{fw}}+c_{\mathrm{up},\mathrm{bk}}\right)}{f_{\mathrm{AP}}}, \end{array}$$

where outage probability in uplink transmission process leads an average of $\left(\varepsilon_{s_{k},\mathrm{tr}}\cdot Q_{s_{k}}\right)$ become unusable. After that, AP sends the backward middle results to user device with latency

(18) $$\begin{array}{c} D_{s_{k},\mathrm{tr},\mathrm{bk}}^{*}=\frac{\left(1-\varepsilon_{s_{k},\mathrm{tr}}^{*}\right)\cdot Q_{s_{k}}\cdot b_{\mathrm{mid},\mathrm{bk}}}{R_{n,\mathrm{dn}}^{*}}. \end{array}$$

Then, user device performs local backward propagation with computing latency

(19) $$\begin{array}{c} D_{s_{k},\mathrm{lc},\mathrm{bk}}^{*}=\frac{{\left(1-\varepsilon_{s_{k},\mathrm{tr}}^{*}\right)}^{2}\cdot Q_{s_{k}}\cdot c_{\mathrm{lw},\mathrm{bk}}}{f_{s_{k}}^{*}}, \end{array}$$

where outage probability in downlink transmission process leads another $\varepsilon_{s_{k},\mathrm{tr}}^{*}$ rate of unstable data. The above process iterates $I_{s_{k}}$ times. Finally, user device upload lower layers to AP with latency

(20) $$\begin{array}{c} D_{s_{k},\mathrm{tr},\mathrm{upld}}^{*}=\frac{b_{\mathrm{lw}}}{R_{n,\mathrm{up}}^{*}} \end{array}$$

Therefore, the total latency with one device is

(21) $$\begin{array}{cc} D_{s_{k}}^{*}= & D_{s_{k},\mathrm{tr},\mathrm{ofld}}^{*}+I_{s_{k}}\cdot \left(D_{s_{k},\mathrm{lc},\mathrm{fw}}^{*}+D_{s_{k},\mathrm{tr},\mathrm{fw}}^{*}+D_{s_{k},\mathrm{AP}}^{*}+D_{s_{k},\mathrm{tr},\mathrm{bk}}^{*}+D_{s_{k},\mathrm{lc},\mathrm{bk}}^{*}\right)\\ \; & +D_{s_{k},\mathrm{tr},\mathrm{upld}}^{*} \end{array}$$

The total latency of SL training process with all participate devices is

(22) $$\begin{array}{c} D_{\mathrm{total}}^{*}=\sum_{k}D_{s_{k}}^{*} \end{array}$$

### 3.3. Proposed Transformer-Based Pre-Validation Model

#### 3.3.1. Overall Architecture

To accurately estimate SL training latency and divergence, a pre-validation model needs to simultaneously model the inter-node relationships, the relationships between different features and estimate output, and the unknown or inaccurate parameters, which is challenging work. In detail, first, the data correlation $C_{s_{k},s_{k+1}}^{⋎}\;\left(k=1,\cdots ,K-1\right)$ between adjacent participating devices greatly influence the overall training divergence, according to Equation (13). Second, the relationships between network states (such as data similarities, wireless channel quality, bit-size of lower layers $b_{\mathrm{lw}}$, etc.) and network resource allocation variables (such as size of mini-batch $Q_{s_{k}}$, training iteration $I_{s_{k}}$, wireless transmitting powers $P_{n}$, etc.) with the training divergence and latency need to be intelligently and automatically established to realize automatic network management in DTN, where the relationships are obviously complex according to Equations (13) and (22). Third, the unknown parameters (such as divergence parameter $\mu_{s_{k}}^{⋎}$) and inaccurate parameters (such as root square variant $\sigma_{n}^{*}$ of channel gain) need to learn without collecting their accurate values.

**Figure 2:**
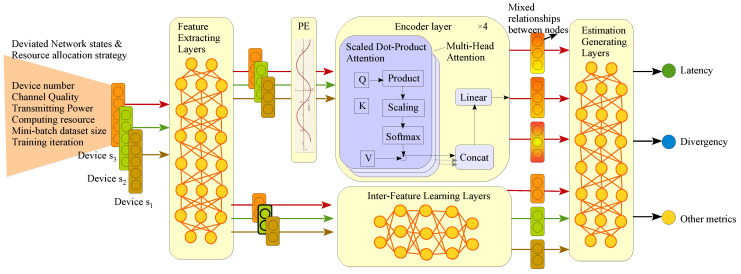
Proposed TransNeural algorithm by combining encoder with neural network.

To do this, we propose a TransNeural algorithm, which combines transformer with neural network to automatically learn the acquired models. The whole architecture is shown in Figure 2. First, we use neural-based feature extracting layers to automatically classify which group of features needs to model their relationships between different devices, and which group of features needs to model their inner-relationship. Then, we design encoder-based line to learn the inter-node relationships and neural-based line to learn inter-feature relationships, respectively. Finally, the outputs of two lines are aggregated using neural network to further learn the complex relationship between two lines with the estimate outputs. In addition, thanks to the added neural network in different positions, the model can learn the unknown or inaccurate parameters automatically.

The proposed TransNeural algorithm can be expressed as a function

(23) $$\begin{array}{cc} \; & \left\{D_{i},\ln\left(\varepsilon_{i}\right)\right\}=\mathrm{TransNeural}\left(\left[x_{i,s_{1}};x_{i,s_{2}};\cdots ;x_{i,s_{K}}\right]\right)\\ \; & =\mathrm{TransNeural}\left(\left[\begin{array}{cccccc} s_{1} & H_{s_{1}}^{*} & P_{s_{1}} & f_{s_{1}}^{*} & Q_{s_{1}} & I_{s_{1}}\\ s_{2} & H_{s_{2}}^{*} & P_{s_{2}} & f_{s_{2}}^{*} & Q_{s_{2}} & I_{s_{2}}\\ \vdots & \vdots & \vdots & \vdots & \vdots & \vdots \\ s_{K} & H_{s_{K}}^{*} & P_{s_{K}} & f_{s_{K}}^{*} & Q_{s_{K}} & I_{s_{K}} \end{array}\right]\right), \end{array}$$

where all of the elements are mean normalized. The loss function is defined as

(24) $$\begin{array}{c} \mathrm{Loss}=\frac{1}{B}\sum_{i}{\left(\frac{D_{i}-D_{\mathrm{real}}}{D_{\max}-D_{\min}}+\frac{\ln\left(\varepsilon_{i}\right)-\ln\left(\varepsilon_{\mathrm{real}}\right)}{\ln\left(\varepsilon_{\max}\right)-\ln\left(\varepsilon_{\min}\right)}\right)}^{2}, \end{array}$$

where B is the size of training batch-size for the TransNeural algorithm.

#### 3.3.2. Feature Extracting Layers

Feature extracting layers aim to automatically project different features into two groups, deciding which group of features are highly combined with inter-node relationships (such as device number), and which contribute to inter-feature learning for establishing complex relationships between network strategy and performances. Elements in different groups may overlap. We use full connected layers to construct such feature extracting layers, which can be expressed as a function by

(25) $$\begin{array}{cc} \; & \left\{\mathrm{Linear}\left(\left[\begin{array}{ccc} s_{1} & Q_{s_{1}} & I_{s_{1}}\\ s_{2} & Q_{s_{2}} & I_{s_{2}}\\ \vdots & \vdots & \vdots \\ s_{K} & Q_{s_{K}} & I_{s_{K}} \end{array}\right]\right),\mathrm{Linear}\left(\left[\begin{array}{cccc} s_{1} & H_{s_{1}}^{*} & \cdots & I_{s_{1}}\\ s_{2} & H_{s_{2}}^{*} & \cdots & I_{s_{2}}\\ \vdots & \vdots & \ddots & \vdots \\ s_{K} & H_{s_{K}}^{*} & \cdots & I_{s_{K}} \end{array}\right]\right)\right\}\\ \; & =\mathrm{FtrEtrL}\left(\left[\begin{array}{cccccc} s_{1} & H_{s_{1}}^{*} & P_{s_{1}} & f_{s_{1}}^{*} & Q_{s_{1}} & I_{s_{1}}\\ s_{2} & H_{s_{2}}^{*} & P_{s_{2}} & f_{s_{2}}^{*} & Q_{s_{2}} & I_{s_{2}}\\ \vdots & \vdots & \vdots & \vdots & \vdots & \vdots \\ s_{K} & H_{s_{K}}^{*} & P_{s_{K}} & f_{s_{K}}^{*} & Q_{s_{K}} & I_{s_{K}} \end{array}\right]\right) \end{array}$$

#### 3.3.3. Positional Encoding Layer

In the SL training task, the device participation sequence greatly influences the training performance, because the initial training divergence on a new device largely depends on its data similarity with the previous training device. Therefore, we need to take the device sequence into consideration to model the relationship between strategies and SL performance. Thus, a positional encoding layer is introduced before the encoder layers. Consistent with classical settings, the expression of positional encoding is based on sine and cosine functions

(26) $$\begin{array}{cc} \; & {\mathrm{PE}}_{\left(k,2i\right)}=\sin\left(k/10,000^{2i/d_{\mathrm{model}}}\right)\\ \; & {\mathrm{PE}}_{\left(k,2i+1\right)}=\cos\left(k/10,000^{2i/d_{\mathrm{model}}}\right) \end{array}$$

where *k* is the participating sequence of a device, $d_{\mathrm{model}}$ is the input dimension of encoder layer, and *i* denotes different input neural of the encoder layer.

#### 3.3.4. Encoder Layers

The encoder layers use the classical components in encoders, including K,Q,V matrices to learn the inter-node relationships by

(27) $$\begin{array}{c} \mathrm{Attention}\left(Q,K,V\right)=\mathrm{softmax}\left(\frac{QK^{T}}{\sqrt{d_{\mathrm{model}}}}\right)V \end{array}$$

We apply multi-head attentions to learn different kind of relationships between devices by

(28) $$\begin{array}{c} \mathrm{MultiHead}\left(Q,K,V\right)=\mathrm{Concat}\left({\mathrm{head}}_{1},\cdots ,{\mathrm{head}}_{h}\right)W^{O},\\ \mathrm{where}\;{\mathrm{head}}_{i}=\mathrm{Attention}\left(QW_{i}^{Q},KW_{i}^{K},VW_{i}^{V}\right) \end{array}$$

Finally, the encoder layer can expressed as

(29) $$\begin{array}{c} \left\{\mu_{s_{k}}Q_{s_{k}}I_{s_{k}},C_{s_{k}}Q_{s_{k}}I_{s_{k}}\right\}=\underset{\mathrm{Modeling}\;\mathrm{data}\;\mathrm{similarities}\;C_{s_{k}}\;\mathrm{and}\;\mathrm{divergency}\;\mathrm{parameter}\;\mu_{s_{k}}.}{\underset{︸}{\mathrm{EncoderL}\left(\left[\begin{array}{ccc} s_{1} & Q_{s_{1}} & I_{s_{1}}\\ s_{2} & Q_{s_{2}} & I_{s_{2}}\\ \vdots & \vdots & \vdots \\ s_{K} & Q_{s_{K}} & I_{s_{K}} \end{array}\right]\right)}} \end{array}$$

#### 3.3.5. Inter-Feature Learning Layers

In the second line, we design inter-feature learning layers to non-linearly produce some key elements for estimating SL performance based on a part of the features. Considering that neural networks are inherently good at extracting the features of different levels in their layers to finally establish the complex relationships between input and output, we simply use fully connected neural network to construct the inter-feature learning layers. That is, in our scenario, the inter-feature learning layers can output wireless outage probability based on the input channel quality, and output SL latency based on the input transmitting power, computing resources, etc. At the same time, it can automatically modify the error in inaccurate computing resource and channel gain based on the device number. The function can be expressed as

(30) $$\begin{array}{c} \left\{D_{i},\left[\varepsilon_{s_{1},\mathrm{tr}},\varepsilon_{s_{2},\mathrm{tr}},\cdots ,\varepsilon_{s_{K},\mathrm{tr}}\right]\right\}=\underset{\mathrm{Modeling}\;\mathrm{relationships}\;\mathrm{and}\;\mathrm{learn}\;\mathrm{accurate}\;H_{s_{k}},f_{s_{k}}.}{\underset{︸}{\mathrm{InFtrL}\left(\left[\begin{array}{cccccc} s_{1} & H_{s_{1}}^{*} & P_{s_{1}} & f_{s_{1}}^{*} & Q_{s_{1}} & I_{s_{1}}\\ s_{2} & H_{s_{2}}^{*} & P_{s_{2}} & f_{s_{2}}^{*} & Q_{s_{2}} & I_{s_{2}}\\ \vdots & \vdots & \vdots & \vdots & \vdots & \vdots \\ s_{K} & H_{s_{K}}^{*} & P_{s_{K}} & f_{s_{K}}^{*} & Q_{s_{K}} & I_{s_{K}} \end{array}\right]\right)}} \end{array}$$

#### 3.3.6. Estimation Generating Layer

Two lines are finally combined in the fully connected layers, which would jointly consider the inter-node relationships and inter-feature relationships to produce the final estimated system performance, i.e., the SL training latency and accuracy in this scenario.

## 4. Simulation

In this section, we evaluate the proposed TransNeural algorithm in Python 3.7.6. We suppose that there are N(N∈[10,30]) devices access to an AP, where the path loss model is $\left(38.46+20\log_{10}\left(d\right)\right)$ [25], *d* is the distance between device and AP. The communication frequency is 2.6 GHz. Each device has a unique identity number from 1 to *N*, which is uniquely projected to a virtual device with that number in DTN. In addition, each device has a series of unique characteristics, which are unknown and about to be automatically learned by DTN models. Their values are randomly set as follows: distance $d_{k}^{*}\in \left[10,900\right]$ m, average reporting deviation on distance ${\Delta d_{k}}^{⋎}\in \left[1,10\right]$ m, average deviation between allocated computing resource and actual provided computing resource ${\Delta f_{k}}^{⋎}\in \left[0.1,1\right]$ GHz, divergence related parameter $\mu_{k}^{⋎}\in \left[1,10\right]$, each element in data similarity vector $C_{k}^{⋎}\in {\left\{[1,5]\right\}}^{N}$. From the parameter settings, it can be seen that the range of value for every parameter is obviously large, with the aim to include as many as network conditions as possible. Notice that, in an SL training process, not all devices will be selected to join the training process. However, the DTN model needs to learn the characteristics of all device to better estimate the training performance for every given resource allocation strategies. Without causing ambiguity, we use SL divergence to replace SL convergence in the simulation section, to make analysis more clearly. Other parameters are given in Table 2.

Details of model in TransNeural algorithm are setting as follows. The dimension of input array is 5, which is the feature dimension of one device. The structure of the feature extracting layer is 64×2, whose first layer and second layer take ReLu function and Linear function as the activation functions, respectively. The encoder block has four encoder layers, where each layer has eight heads for multi-head attention, and the dimension of input vector is 64. The structure of inter-feature learning layer is 64×2, whose layers take ReLu function as the activation functions. The structure of estimation generating layer is 64×2, whose first layer and second layer take ReLu function and Linear function as the activation functions, respectively.

The baselines are set as follows:

- **Equation-based algorithm:** this algorithm estimates latency and divergency based on the equations in Section 3.1 and Section 3.2.
- **LSTMNeural algorithm:** We merge the long short-term memory (LSTM) and neural network to form a LSTMNeural algorithm. That is, in the architecture of proposed TransNeural algorithm, the PE layer and encoder layer are replaced by an LSTM. The input dimension of the LSTM is 16. The architecture of its hidden layer is 32×4.

**Figure 3:**
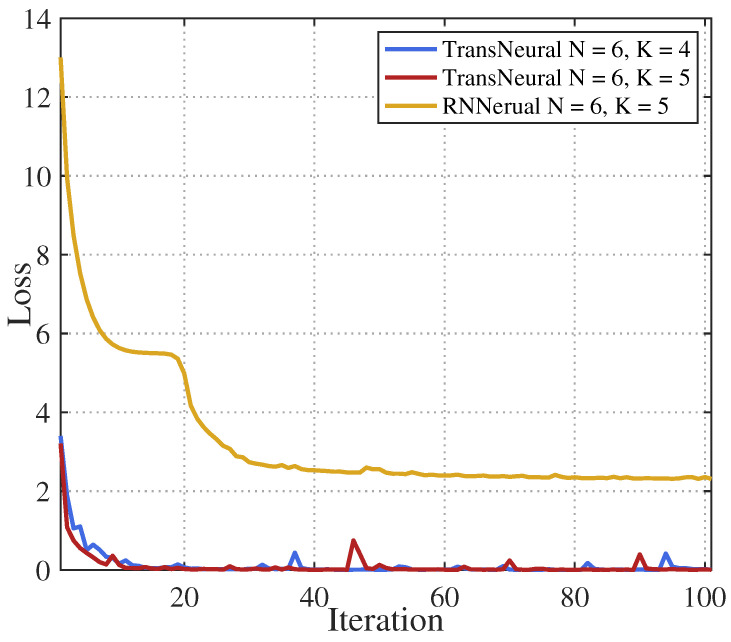
Learning convergence of proposed TransNeural algorithm.

Figure 3 gives learning convergence of the LSTMNeural and proposed TransNeural algorithm. The figure indicates that the TransNeural algorithm converges faster than the LSTMNeural algorithm, and can acquire a lower loss. The reason lies in the fact that, on each data sample, LSTM can only learn the data correlations between adjacent participate devices, while transformer can learn the data correlations between all of the participate devices. Therefore, TransNeural algorithm has a much higher learning efficiency to estimate network performance. In addition, the figure indicates that the TransNeural algorithm converges fast under different size of SL participant group.

**Figure 4:**
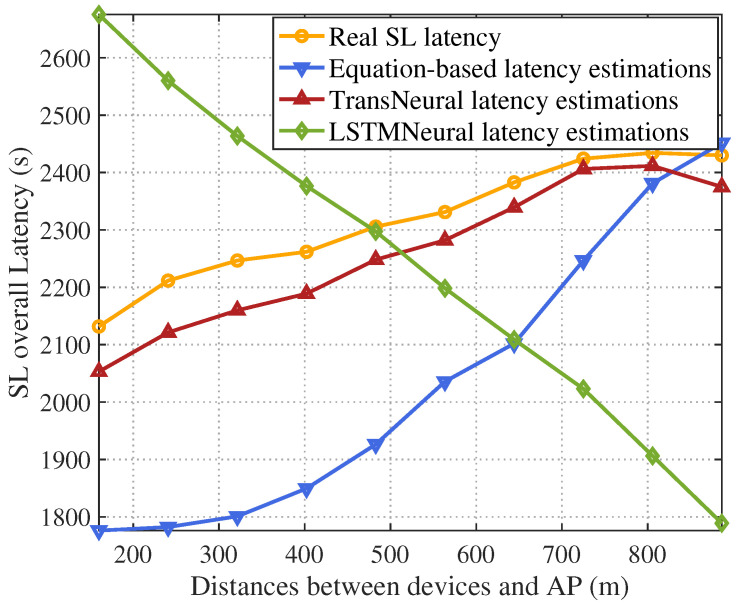
SL training latency vs. distances between devices and AP.

**Figure 5:**
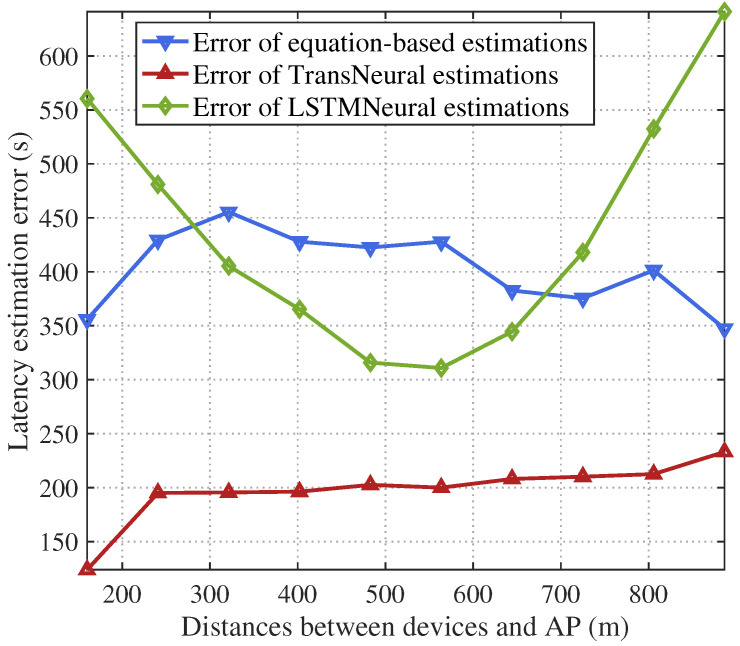
Estimation error of SL training latency vs. distances between devices and AP.

Figure 4 and Figure 5 give the total SL latency and estimation error with growing average distance between selected user devices and AP. In general, the wireless transmission rate will first drop as the distance grows. Considering the wireless transmission happens frequently in each forward and backward propagation process, the transmission delay will significantly influence the total SL training delay, which leads the total SL delay increases as the distance grows. From the figure, since the DTN may not acquire accurate CSI, latency estimation based on traditional equation-based method will have a high error, especially under larger distance where CSI error becomes sensitive for latency estimation. In comparison, the proposed TransNeural algorithm has a stable error with distance growing, which stays under 240 s per omit-table because the total error is 2100–2300 s. However, LSTMNeural has a large estimation error because of its low learning efficiency.

**Figure 6:**
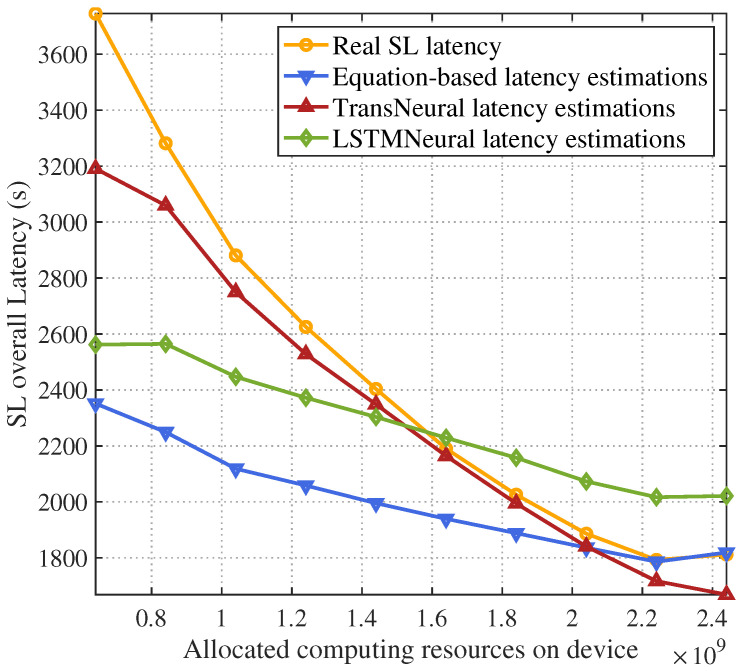
SL training latency vs. computing resources on devices.

**Figure 7:**
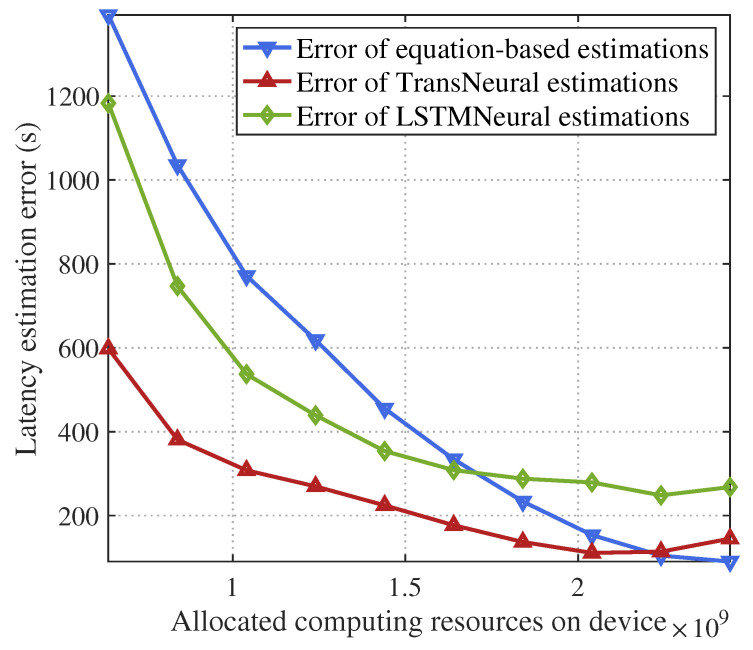
Estimation error of SL training latency vs. computing resources on devices.

Figure 6 and Figure 7 give the total SL latency and estimation error with a growing allocated computing resources on devices. Because the user device needs to use their allocated computing resources to compute forward and backward propagation of lower layers in SL target models, the computing latency will decrease with the increasing allocated computing resources, which leads the total latency decrease. However, because of user misreport information, the real allocated computing resources may be smaller than the amount of allocated resource in network strategies, which can not be modeled in traditional equation-based latency estimation methods, leading to estimation errors. In comparison, the proposed TransNeural algorithm and LSTMNeural algorithm can automatically learn these misreport behaviors from historical data, thus better estimate the real latency than traditional methods. Moreover, the TransNeural outperforms LSTMNeural because of high learning efficiency.

**Figure 8:**
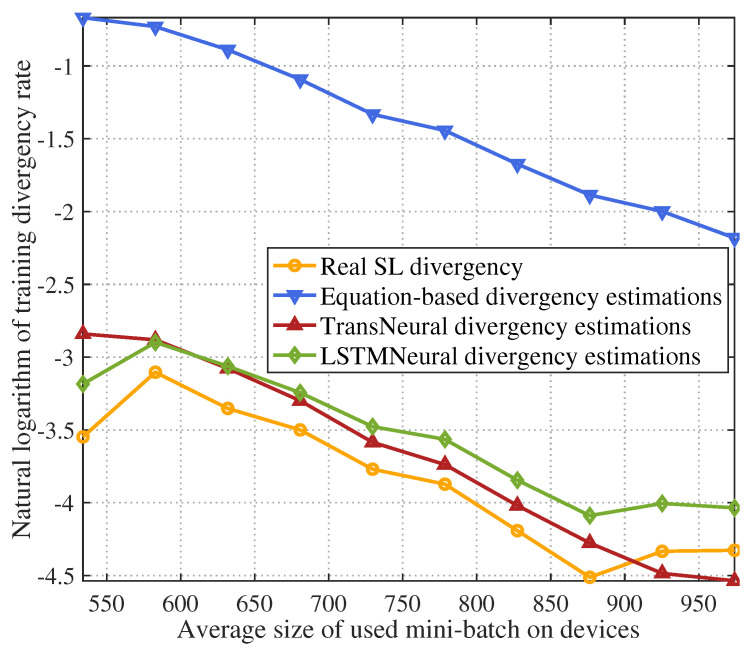
SL training divergence rate vs. average size of mini-batch used for training on devices.

Figure 8 gives the natural logarithm of the training divergence with growing average size of mini-batch used for training on user devices. The divergence drops as the size of mini-batch grows. Since the equation-based algorithm cannot learn the data correlations between device and takes the average correlation value to estimate divergence, its estimation has a large error compared with two other algorithms. In comparison, the proposed TransNeural algorithm can accurately predict the divergency under various size of mini-batch, thanks to its high learning ability. In addition, the figure indicates that the LSTMNeural algorithm has a higher estimation error than the proposed TransNeural algorithm. This is because that the LSTM algorithm cannot leverage dataset to learn data correlations between different devices efficiently compared with the transformer algorithm, as discussed earlier.

**Figure 9:**
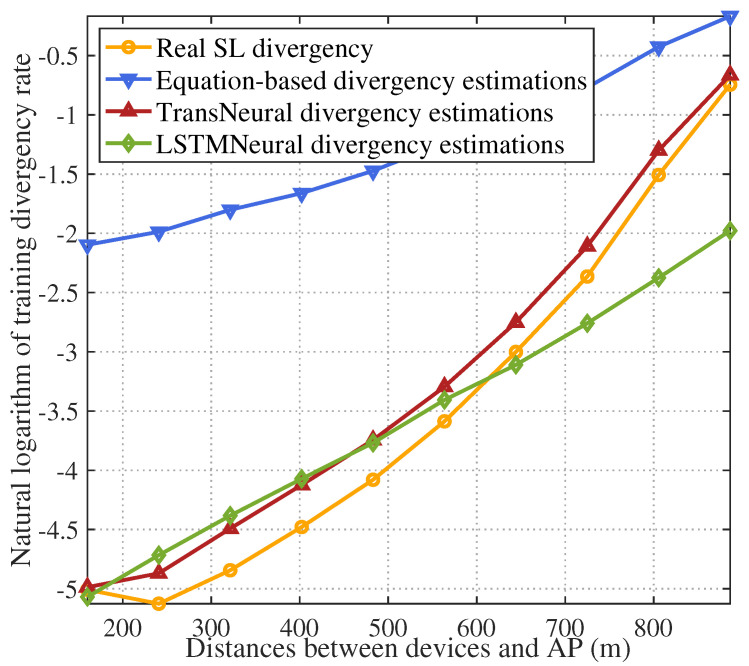
SL training divergence rate vs. distances between devices and AP.

Figure 9 gives the natural logarithm of training divergence with growing average distances between selected devices and AP. As distance grows, outage probability in wireless transmission process increases. It greatly influences the SL training divergence considering the dense wireless transmission process exists in SL training, which is also proved in the figure. In traditional equation-based algorithm, inaccurate CSI leads to a high divergency estimation error compared with the other two algorithm, which could eliminate error because of integrated neural network.

**Figure 10:**
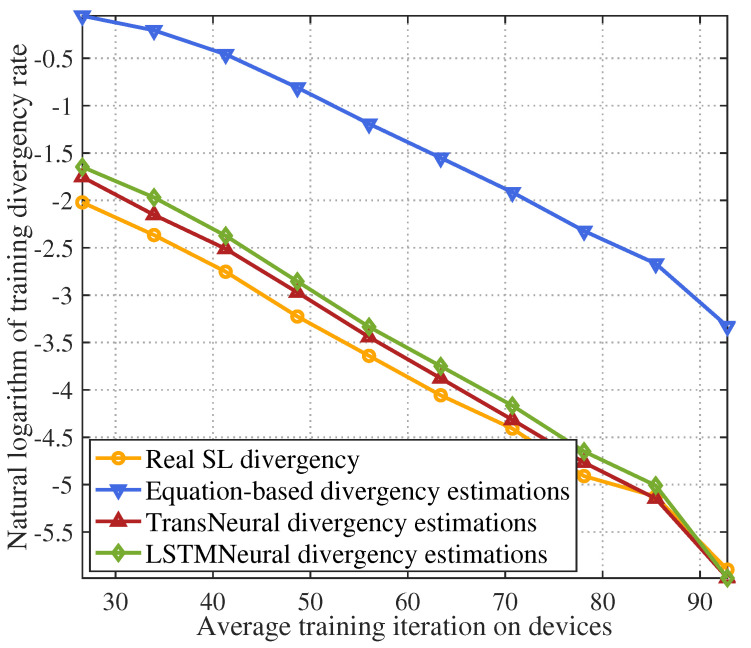
SL training divergence rate vs. average training iterations among devices.

Figure 10 gives the natural logarithm of training divergence with growing average training iterations among participating devices. In general, the divergence decreases as the training iteration increasing. The equation-based algorithm has a higher estimating error compared with two other algorithms because of lacking accurate data correlation parameters. Moreover, the proposed TransNeural algorithm outperforms the LSTMNeural algorithm because of high learning efficiency on given dataset.

Table 3 compares the estimation accuracy on SL training latency and divergence under different algorithms. As for the SL latency, the proposed TransNeural algorithm increases the estimating accuracy by 9.3% and 6.5% compared with the equation-based algorithm and LSTMNeural algorithm, respectively. As for the SL convergence, the proposed TransNeural algorithm increases estimating accuracy by 22.41% and 2.34% compared with the equation-based algorithm and LSTMNeural algorithm, respectively. The table proves that the proposed algorithm can effectively improve the estimation accuracy for various types of SL metrics.

Table 4 and Table 5 verify the algorithm scalability. In detail, Table 4 shows that the proposed TransNeural algorithm remains a high latency estimation accuracy with growing number of devices. The estimation accuracies under different numbers of devices exhibit fluctuations, since different data distributions may result in different degrees of learning difficulties. Fortunately, they are all higher than 90%, which verifies the scalabilities of the proposed TransNeural algorithm. Similarly, Table 5 shows that the proposed TransNeural algorithm remains a high convergence estimation accuracy with growing number of devices. That is, all of the convergence estimation accuracies under different number of devices are high than 95%, which again proves the well scalability of proposed TransNeural algorithm.

## 5. Conclusions

The paper closes the gap of studying the pre-validation environment for SL training tasks. It proposes a TransNeural algorithm to estimates the latency and divergency of SL training process under given resource allocation solution. In detail, the TransNeural algorithm integrates the transformer and neural network to collaboratively learn data similarities, complex relationships between SL performance (latency, divergency, etc.) and participants selections, wireless/computing resource allocation, and the reported deviation on wireless channels and available computing resources. Simulation shows the proposed TransNeural algorithm can effectively improve the estimating accuracy by 9.3% on latency and 22.4% on divergency compared with traditional equation-based algorithms.

## Figures and Tables

**Table 1:** Index of key notations.

Notation	Description
$Q_{n},Q_{n}^{\mathrm{sum}}$	Dataset on the *n*-th device, size of dataset on the *n*-th device
D,O,G,E,Z	DTN notation, network management module, network strategy module, pre-validation environment and network database in DTN
$f_{\mathrm{av},n},f_{\mathrm{av},n}^{*};f_{n},f_{n}^{*}$	Reported and real available computing resources, expected and real computing resources allocating strategy
$R_{n,\mathrm{dn}},R_{n,\mathrm{up}};R_{n,\mathrm{dn}}^{*},R_{n,\mathrm{up}}^{*}$	Expected transmission rate in downlink and uplink; Real transmission rate in downlink and uplink
$\varepsilon_{s_{k},\mathrm{tr}}^{*},\varepsilon_{s_{k},\mathrm{cp}}^{⋎},\varepsilon_{s_{k}}^{⋎}$	Outage probability, computing divergence, final divergence on the $s_{k}$-th device
$C^{⋎},I_{s_{k}}$	Data correlation matrix, training iteration
$b_{\mathrm{lw}},b_{\mathrm{up}},b_{\mathrm{mid},\mathrm{fw}},b_{\mathrm{mid},\mathrm{bk}}$	Bit-size of lower and upper layers in target model, bit-size of middle parameter in forward and backward propagation

**Table 2:** Parameter settings.

Parameter	Value	Parameter	Value
$Q_{k}$	$\left[100,1000\right]$	$I_{k}$	$\left[5,100\right]$
$f_{k}$	$\left[0.1,2\right]$ G cycles/s	$f_{B}$	100 G cycles/s
*B*	10 MHz	*K*	64
$N_{0}$	$10^{-20.4}$	$H_{L}^{k}$	$140\times 10^{-6}$
$P_{k}$	$\left[0.01,2\right]$ W	$P_{B}$	88 W
$b_{\mathrm{mid},\mathrm{fw}}$	1 Mbits	$b_{\mathrm{mid},\mathrm{bk}}$	1 Mbits
$c_{\mathrm{up},\mathrm{fw}}$	100 M cycles	$c_{\mathrm{up},\mathrm{bk}}$	100 M cycles
$c_{\mathrm{lwer}}^{\mathrm{forward}}$	1 M cycles	$c_{\mathrm{lw},\mathrm{bk}}$	1 M cycles
$b_{\mathrm{lw}}$	1 Gbits		

**Table 3:** Comparison of deviant ratio for estimated latency and divergence in SL training process.

	Latency Estimation (s)	Accuracy
Actual SL latency	2316.0	/
TransNeural algorithm	2114.8	91.3%
LSTMNeural algorithm	1961.3	84.7%
Equation-based algorithm	1897.9	82.0%
	**Convergence Estimation**	**Accuracy**
Actual SL convergence	97.82%	/
TransNeural algorithm	95.89%	98.03%
LSTMNeural algorithm	93.60%	95.69%
Equation-based algorithm	73.97%	75.62%

**Table 4:** Accuracy of latency estimation under different participant scales.

N=10,K=5	N=15,K=10	N=20,K=15	N=25,K=20	N=30,K=25
91.3%	93.8%	98.4%	99.5%	97.3%

**Table 5:** Accuracy of convergence estimation under different participant scales.

N=10,K=5	N=15,K=10	N=20,K=15	N=25,K=20	N=30,K=25
98.0%	99.1%	99.7%	96.0%	97.2%

## Data Availability

Data can be found in the article.
